# Targeted nanocomposite delivery system of amygdalin using chitosan/reduced graphene oxide-zinc oxide/hyaluronic acid for treatment of head and neck squamous cell carcinoma

**DOI:** 10.1186/s12903-026-07943-1

**Published:** 2026-03-25

**Authors:** Sarah Ibrahim Baghdady, Sally A. Sabra, Asmaa Badawy Darwish, Abeer Salama, Elbadawy A. Kamoun, Seham A. Hanafy

**Affiliations:** 1 Dental Biomaterials Department, Faculty of Dentistry, Alexandria University, Champolion St., Azarita, Alexandria 21526, Egypt; 2 Department of Biotechnology, Institute of Graduate Studies and Research, Alexandria University, Alexandria, 21526 Egypt; 3 Pharmaceutical Technology Department, National Research Centre, Cairo 12622, Egypt; 4 Pharmacology Department, Medical Research and Clinical Studies Institute, National Research Centre, Cairo 12622, Egypt; 5 Department of Chemistry, College of Science, King Faisal University, Al- Ahsa, 31982 Saudi Arabia; 6 Polymeric Materials Research Department, Advanced Technology and New Materials Research Institute (ATNMRI), City of Scientific Research and Technological Applications (SRTA-City), New Borg Al-Arab 21934, Alexandria, Egypt

**Keywords:** Amygdalin, Chitosan, Graphene oxide, Hyaluronic acid, Zinc oxide, Head and neck cancer, Squamous cell carcinoma, Targeting, Controlled release

## Abstract

**Background:**

Head and neck squamous cell carcinoma (HNSCC) is a major and growing public health problem due to its global high incidence and mortality. Most conventional treatments have adverse side effects due to lack of selectivity and targetabiliy, which has increased the demand for more precise and safe alternatives. Amygdalin (AMG) is a plant-derived cyanoglycoside with a potent anticancer effect, however, its therapeutic use remains controversial due to safety concerns.

**Objective:**

Nanotechnology can improve the targeted delivery of AMG while reducing its drawbacks. This study aimed to develop a nanocomposite comprised of chitosan (CS), reduced graphene oxide (rGO), zinc oxide nanoparticles (ZnO NPs), and hyaluronic acid (HA) to serve as a nanocomposite for controlled release and targeted delivery of AMG.

**Methods:**

AMG-loaded (CS/rGO-ZnO/HA) nanocomposite was prepared by the ion gelation technique using tripolyphosphate (TPP) as a crosslinker. Blank and AMG-loaded nanocomposites were characterized using FTIR, XRD, TEM, SEM, DSC, and TGA. In vitro drug release was conducted via the dialysis bag method. In vitro anticancer effect of the prepared nanocomposite was evaluated via cell viability, wound scratch, and cellular uptake assays.

**Results:**

AMG-loaded nanocomposite manifested a particle size of 180.2 ± 2.13 nm with a negative surface charge of -36.5 ± 4.96 mV, high encapsulation efficiency (90.81%±1.23), and pH-dependent controlled release. Moreover, AMG-loaded nanocomposite exhibited a much lower IC_50_ value in comparison to free AMG when examined against A-341 cells. In addition, AMG-loaded nanocomposite demonstrated improved anti-migratory effect, associated with enhanced cellular uptake due to its active targeting potential.

**Conclusion:**

AMG-loaded nanocomposite could be considered a promising targeted nanoplatform for managing head and neck squamous cell carcinoma.

**Supplementary Information:**

The online version contains supplementary material available at 10.1186/s12903-026-07943-1.

## Introduction

Cancer represents a significant global health issue, evidenced by the persistent rise in cases in recent decades, as reported by the International Agency for Research on Cancer [1, 2]. Approximately 90% of epithelial malignancies in the head and neck region are classified as squamous cell carcinoma. Head and neck squamous cell carcinoma (HNSCC) is the seventh most prevalent cancer globally. Annually, over 940,000 new cases are identified, with around 455,000 fatalities, according to GLOBOCAN 2022 data [2]. HNSCC usually metastasizes and/or recurs, with around 50% of patients showing lymph node involvement at diagnosis [3]. The incidence of HNSCC is supposed to increase significantly, with an anticipated annual rise of 30% by 2030 [3]. While surgical excision is the gold standard for invasive squamous cell carcinoma (SCC), radiotherapy serves as a vital alternative for patients who are ineligible for surgery. There is a continuous progression to develop new therapies, including immunotherapies such as immune checkpoint inhibitors (e.g., Cemiplimab) and epidermal growth factor receptor (EGFR) inhibitors [4]. These treatments indeed show promise for cancer management; however, each modality presents significant drawbacks [5].

Herbal medicine presents a promising frontier for cancer treatment owing to its pharmacological efficacy, favorable safety profiles, and cost-effectiveness. Over the past three decades, nearly 80% of cancer therapeutics approved by the FDA have derived from, or inspired by, natural constituents [6]. Consequently, exploring these bioactive agents is essential for the development of next-generation chemopreventive and chemotherapeutic drugs.

Amygdalin (AMG, Vitamin B17); a cyanogenic glycoside naturally abundant in the kernels of the *Rosaceae* family (particularly bitter almonds and apricots), is increasingly recognized for its pharmacological efficacy and selective toxicity in cancer therapy [7, 8]. It can arrest the cell cycle and induce apoptosis in malignant cells. Moreover, it exhibits antioxidant capabilities that could potentially reduce oxidative stress in cancer cells [9]. Although amygdalin has demonstrated a potential efficacy in the treatment of various malignancies [10–12], the risk of cyanide toxicity upon oral administration hinders its clinical application [13]. This critical constraint, in addition to its poor solubility, instability, and rapid elimination, has led researchers to develop drug delivery systems (DDSs) to circumvent toxicity and enhance the therapeutic efficacy of AMG [7–10, 14–16].

Chitosan (CS) as s a stimuli-responsive polymer can facilitate targeted drug delivery and controlled release. Moreover, its inherent biocompatibility, biodegradability, and mucoadhesive properties make it a promising candidate for HNSCC therapy [17]. HNSCC cells characteristically overexpress CD44 receptors, which also serve as critical markers for resistant cancer stem cells (CSCs) [18]. These receptors function as a primary receptor for hyaluronic acid (HA), and hence targeting this signaling pathway represents a promising strategy to disrupt tumor development and overcome CSC-mediated resistance [19].

Graphene and its derivatives, including graphene oxide (GO) and reduced graphene oxide (rGO), are intriguing drug delivery platforms due to their large surface area, unique chemical structures, and innate cytotoxicity against certain malignancies [20, 21]. Functionalized graphene biocomposites integrated with chitosan and/or hyaluronic acid can improve the overall targeting, therapeutic efficacy, and biocompatibility of these composites [22]. Furthermore, zinc oxide nanoparticles (ZnO NPs) can selectively induce reactive oxygen species (ROS) and oxidative stress in malignant cells while remaining biocompatible with healthy tissues. Mechanistic evidence suggests that co-administering zinc with amygdalin synergistically enhances antineoplastic efficacy by augmenting the apoptotic response in specific cancer cell lines [23, 24].

These nanocomposites, composed of CS, rGO, ZnO NPs, and HA, can harness the synergistic features of their constituents to enhance drug delivery, targeting, and therapeutic efficacy across diverse cancer models [20, 21, 24–26]. While existing literature has explored subsets of these bioactive materials, such as CS/GO/ZnO or CS/HA/ZnO. The concurrent integration of CS, rGO, ZnO NPs, and HA within a single quaternary formulation remains unexplored. This study is the first to develop an advanced pH-responsive, cross-linked nanocomposite (CS/rGO-ZnO/HA) designed to synergistically leverage the pH-responsiveness of CS, the high loading capacity of rGO, the selective toxicity of ZnO NPs, and the CD44-targeted delivery by HA. Furthermore, there is a paucity of literature regarding the therapeutic efficacy of AMG in the context of head and neck squamous cell carcinoma (HNSCC); consequently, a significant knowledge gap remains concerning its safety profile and in vitro bioactivity. To address this, this current study aims to engineer a novel quaternary delivery system to encapsulate AMG, providing a physiologically relevant platform to evaluate its cytotoxicity and therapeutic potential against HNSCC cell lines.

## Materials and methods

### Materials

Amygdalin (AMG; ≥99% purity, cat. no. A20150-5.0) was purchased from Research Product International (RPI; Mt. Prospect, IL, USA). Chitosan (CS; MW > 300 kDa, cat. no. 937657), sodium tripolyphosphate (TPP; cat. no. 238503), sodium hydroxide pellets (NaOH; cat. no. S5881), methanol (cat. no. 48335), Dulbecco’s Modified Eagle Medium (DMEM), fetal bovine serum (FBS), and bovine serum albumin (BSA) were obtained from Sigma-Aldrich (St. Louis, MO, USA). Reduced graphene oxide doped with zinc oxide (rGO-ZnO) was sourced from Nano Gate. Co. (Cairo, Egypt). Hyaluronic acid (HA; MW > 1,000 kDa, 96% purity) was generously provided as a gift by Al-Andulus Pharmaceuticals (Cairo, Egypt). Glacial acetic acid (99% purity) and phosphate-buffered saline (PBS) tablets, and 3- (4, 5-dimethylthiazol-2-yl)-2, 5-diphenyl tetrazolium bromide (MTT) were purchased from Thermo Fisher Scientific (Waltham, MA, USA). Penicillin–streptomycin solutions (100 U/mL) were obtained from BioWhittaker^®^ (Lonza, Verviers, Belgium). The human fibroblast (HSF; ATCC^®^ CRL-2465™) and the human squamous cell Carcinoma (A-431; ATCC^®^ CRL-1555™) cell lines were obtained from the American Type Culture Collection (ATCC) and the Holding Company for Biological Products and Vaccines (VACSERA, Giza, Egypt), respectively. All other reagents were of analytical grade. In vitro experiments were conducted at the Center of Excellence for Research in Regenerative Medicine and Applications (CERMA), Faculty of Medicine, Alexandria University, Egypt.

### Preparation of hyaluronic acid-coated chitosan nanoparticles functionalized with rGO-ZnO (CS/rGO-ZnO/HA)

Chitosan nanoparticles (CSNPs) were prepared using the ionic gelation method [27]. The specific compositions of the various prepared NP formulations are summarized in Table 1. Briefly, chitosan (CS) at different concentrations was dissolved in 100 mL of a 1% (v/v) acetic acid solution until complete dissolution was achieved. The solution pH was adjusted to 5.0 using sodium hydroxide (NaOH) and subsequently passed through a 0.45 μm pore size filter. Simultaneously, rGO-ZnO powder was dispersed in deionized water at a concentration of 20% (w/w) relative to the CS mass [28]. This rGO-ZnO dispersion was added gradually to the CS solution while being magnetically stirred at 700 rpm. The CS solution was subsequently cross-linked via the dropwise addition of a negatively charged tripolyphosphate (TPP) solution (0.166 g dissolved in 10 mL of deionized water). This reaction was maintained under constant magnetic stirring at 700 rpm for 2 h at ambient temperature [29, 30]. Finally, hyaluronic acid (HA) solution was introduced dropwise into the CS/rGO-ZnO nanoparticle suspension in varying proportions to yield the final CS/rGO-ZnO/HA nanocomposite. The resulting mixtures were magnetically stirred for an additional 1 h at 400 rpm to attain colloidal stabilization [31]. Through this systematic approach, six different formulations were successfully developed for further evaluation.

**Table 1:** Different formulations of AMG-loaded (CS/rGO-ZnO/HA) nanocomposite with different optimization factors and responses, including mean entrapment efficiency and physiochemical properties of AMG-loaded nanocomposite

Formulation	CS (mg)	AMG (mg)	CS: HA ratio (wt./wt.)	EE%	Particle size (nm)	PDI	Zeta potential (mV)
F1	250	25	6:1	22.45%±4.23	627.2 ± 3.25	0.455 ± 0.08	-47.1 ± 1.27
F2	250	50	6:1	79.90%±3.26	496.9 ± 3.68	0.593 ± 0.02	-55.3 ± 1.25
F3	250	75	6:1	88.47%±1.96	418.8 ± 3.29	0.429 ± 0.07	-57.1 ± 2.63
F4	500	25	10:1	43.30% ±2.54	359.2 ± 2.36	0.438 ± 0.05	-24.1 ± 2.12
F5	500	50	10:1	85.12%±2.36	272.2 ± 1.22	0.205 ± 0.06	-28.1 ± 3.26
F6	500	75	10:1	90.81%±1.23	180.2 ± 2.13	0.282 ± 0.03	-36.5 ± 4.96

All experiments were conducted in triplicate, and the results were represented as mean ± SD

### Preparation of amygdalin-loaded (CS/rGO-ZnO/HA) nanocomposite

**Fig. 1:**
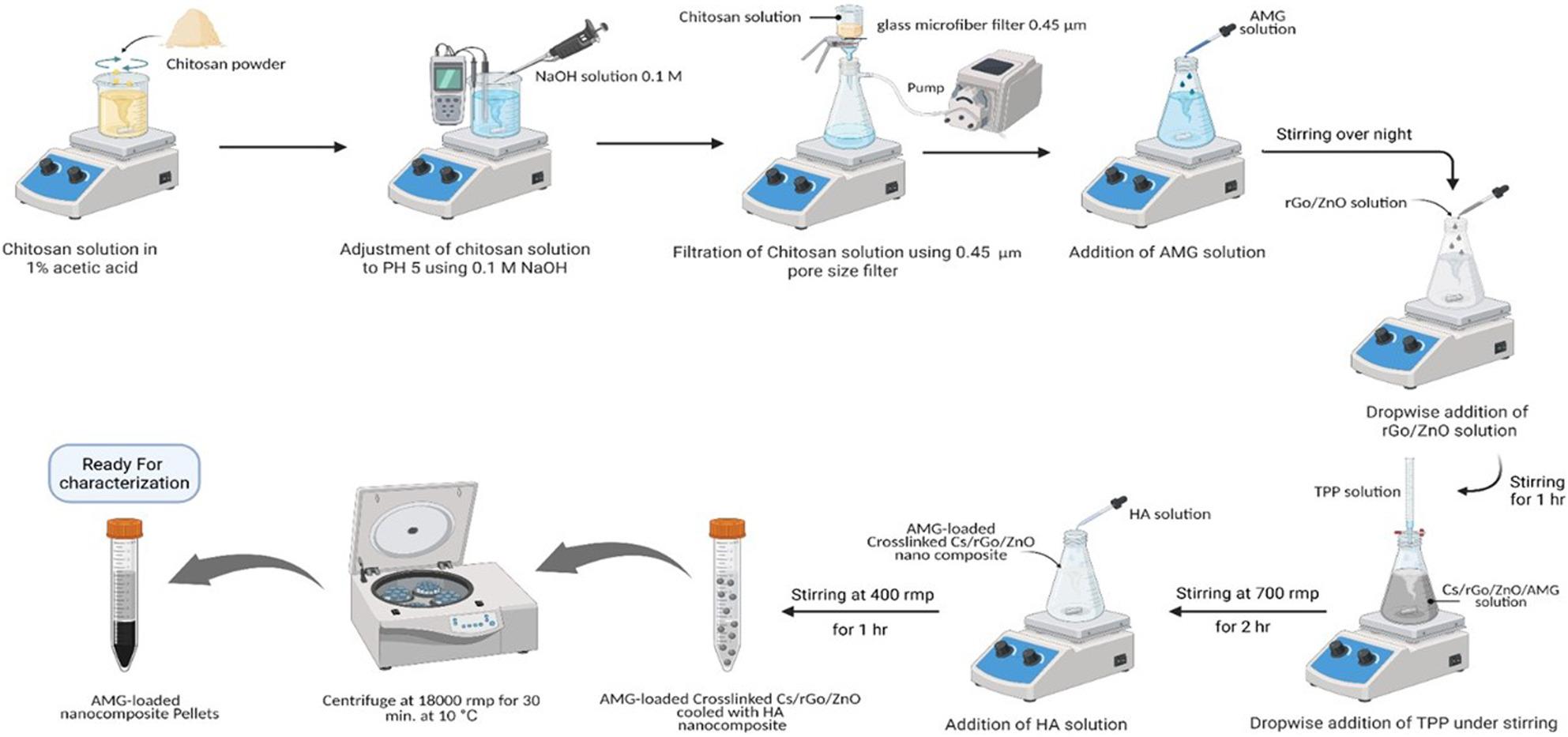
Schematic illustration of the preparation of Amygdalin-loaded (CS/rGO-ZnO/HA) nanocomposite. Created with BioRender.com; Publication ID: UI28OI0DN2

Amygdalin (AMG) stock solutions were prepared by dissolving AMG in distilled water at various concentrations. To prepare the drug-loaded system, the AMG solution was added dropwise to the filtered CS solution and homogenized under continuous magnetic stirring overnight. Following this incorporation, the subsequent synthesis steps were performed exactly as described in the previous section to yield the final nanocomposites (Fig. 1).

### Determination of the entrapment efficiency percentage (EE%)

Free AMG was separated from AMG-loaded (CS/rGO-ZnO/ HA) nanocomposite via centrifugation at 5200 xg and 4 °C for 30 min utilizing a high-speed centrifuge (Sigma Laboratory Refrigerated Centrifuge, Model 3 K-30, Germany). The supernatant was discarded, while the precipitated (CS/rGO-ZnO/HA/AMG) NPs were washed twice with distilled water and re-centrifuged to remove any residuals. The obtained pellets were used to measure the entrapment efficiency (EE%).

Entrapment efficiency (EE%) was assessed by methanol extraction of the encapsulated drug. Methanol was the optimum solvent for this extraction because AMG exhibits high solubility in alcoholic solutions, exceeding 35%. For the extraction, 200 µL of methanol was mixed with 10 mg of pellet and subjected to vigorous vortexing. This process allowed the methanol to disrupt the nanocomposite structure, thereby releasing AMG into the solution. The resulting clear supernatant was carefully collected. The amount of entrapped AMG in the pellet, which contributes to the EE%, was then determined by measuring the drug’s absorbance in the supernatant using a UV-Vis spectrophotometer (UV-2401 PC, Shimadzu Co., Japan) at a fixed maximum wavelength (λ_max_) of 262 nm according to a previously established calibration curve (Stock solution 250 µg/mL, concentrations range 7.8125–125 µg/mL). Methanol served as the reference in the spectrophotometer. All measurements were conducted trice. Finally, the entrapment efficiency (EE %) of the amygdalin-loaded CS/rGO-ZnO/HA nanocomposite was calculated as follows:

1 $$\mathrm{EE}\%\;=\mathrm{Amount}\;\mathrm{of}\;\mathrm{Entrapped}\;\mathrm{AMG}\;/\mathrm{Total}\;\mathrm{Amount}\;\mathrm{of}\;\mathrm{AMG}\;\mathrm{Added}\times\;100$$

### Physicochemical characterization

The average hydrodynamic diameter (particle size; PS) and zeta potential (ZP) of the various nanoformulations were determined via dynamic light scattering (DLS) technique on a Zetasizer Nano ZS (Malvern Instruments Ltd., Worcestershire, UK). All measurements were performed in triplicate at room temperature using a scattering angle of 173 degrees. The optimal formulation was identified by evaluating the encapsulation efficiency (EE%), hydrodynamic diameter (PS), and zeta potential (ZP) of all prepared systems.

### Morphological examination

Scanning electron microscopy (SEM) technique (JEOL JEM-1230, Japan) was utilized to analyze surface morphology and porosity of the constructed AMG-free and AMG-loaded (CS/rGO-ZnO/HA) nanocomposite. Before examination, samples were dispersed using ultrasound and dried in a vacuum oven for one day. Finally, samples were coated with a thin layer of gold to improve image quality. SEM imaging was performed at an accelerating voltage of 20 kV with a fixed working distance of 10 nm to ensure optimal resolution and depth of field.

The morphological characteristics and internal structure of the preparednanocomposites were examined using transmission electron microscopy (TEM) (model JEM-100 S microscope, JEOL, Tokyo, Japan) at a high voltage of 160 kV. Samples were prepared by dispersing AMG-free or AMG-loaded (CS/rGO-ZnO/HA) nanocomposites in 5 mL of distilled water, then one drop of each sample was placed on a carbon-coated grid and left for 1 min to allow adherence to the carbon surface. Samples were finally air-dried for 1 min before microscopic imaging.

### Fourier transform infrared spectroscopy (FTIR)

FTIR spectroscopy (PerkinElmer Life and Analytical Sciences, Shelton, CT, USA) was used to study the interaction between AMG and different components of the developed (CS/rGO-ZnO/HA) nanocomposite. Individual components, including chitosan (CS), hyaluronic acid (HA), amygdalin (AMG), reduced graphene oxide/zinc oxide (rGO-ZnO), as well as AMG-free and AMG-loaded (CS/rGO-ZnO/HA) nanocomposite, were investigated. FTIR samples were prepared by meticulous mixing of approximately 2–3 mg of each test material with 200 mg of spectroscopic- grade anhydrous potassium bromide (KBr) powder. The mixture was finely ground using an agate mortar and pestle under ambient conditions in a controlled environment. Subsequently, the homogeneous mixtures were compressed into transparent discs using a hydraulic press under a pressure of 10 tons for 2 min to eliminate air bubbles and ensure uniform sample distribution throughout the disc matrix. FTIR spectra were recorded for each sample in the range of 4000 –400 cm^− 1^[29].

### X-ray diffraction (XRD)

An X-ray diffractometer (XRD) was used to assess the crystallinity of the AMG loaded in (CS/rGO-ZnO/HA) nanocomposite. The diffraction pattern was obtained at a voltage of 30 kV and a current of 30 mA within the range of 10° < 2θ < 40°, utilizing a step size of 0.02°. Individual components, including CS, HA, rGO-ZnO, and AMG, as well as drug-free and AMG-loaded (CS/rGO-ZnO/HA) nanocomposites, were investigated.

### Differential scanning colorimetry (DSC)

The thermal profiles and phase transitions of each component, including CS, HA, rGO-ZnO, and AMG, as well as drug-free and AMG-loaded (CS/rGO-ZnO/HA) nanocomposite were investigated using Differential Scanning Colorimetry (DSC) (DSC-60, Shimadzu, Tokyo, Japan) equipped with a TA-60 WS thermal analyzer. The instrument was calibrated using indium as a standard reference. The formulated nanoparticle dispersion was lyophilized before DSC examination utilizing a freeze-dryer. The Shimadzu TA-60 software was used to measure thermograms. A known quantity of the lyophilized NPs was scanned in a hermetically sealed aluminum pan. The scanning was conducted at a rate of 5 °C/min with a temperature range of 20–300 °C under nitrogen purging at a flow rate of 25 mL/min.

### Thermogravimetric analysis (TGA)

The thermal stability and decomposition profiles of the raw components (CS, HA, rGO-ZnO, and AMG), and the resulting nanocomposites, both drug -free and AMG-loaded (CS/rGO-ZnO/HA) nanocomposites, were analyzed via TGA using a Q600 thermal analyzer (TA Instruments, New Castle, DE, USA). Samples were heated from 0 to 900 °C at a constant heating rate of 10 °C/min under a protective argon atmosphere [30].

### In vitro drug release study

The dialysis bag assay was used to study the in vitro release characteristics of AMG from the developed nanocomposite in two release media: PBS (pH 7.4) and PBS (pH 5.5) to mimic the tumor acidic microenvironment [32]. The developed AMG-loaded nanocomposite CS/rGO-ZnO/HA/AMG) was resuspended in PBS, then it was subsequently placed into a pre-soaked cellulose dialysis bag (MWCO 8–14 kDa) containing a volume equivalent to 2 mg of AMG. After which, the bag was sealed on both ends. The bags were subsequently positioned in sealed glass containers with 100 mL of the release medium (PBS, pH 7.4 or PBS, pH 5.5). Containers were kept in a shaking water bath (Memmert, SV 1422, Germany) at 100 rpm of rotation and 37 °C. To restore the sink condition, 2 mL of the release medium was withdrawn at preassigned time intervals and compensated with the exact volume of fresh media. The concentration of the released AMG was determined spectrophotometrically at 262 nm. The amount of the released AMG in relation to the initial amount of AMG in the dialysis bag was used to calculate the cumulative % of AMG released. Results were represented as the mean of three values ± SD.

To investigate the mechanism of AMG release from the developed nanocomposite, kinetic analysis of the release data was performed. Multiple kinetic equations were employed to fit the data: the zero-order model (% cumulative drug released versus time), the first-order model (log% cumulative drug remaining versus time), the Higuchi model (% cumulative drug released versus square root of time), and the Korsmeyer–Peppas model, including the percentage of cumulative drug released up to 60% (log% cumulative drug released versus log time). The most fit release model was selected according to the regression coefficient (R^2^) derived from linear regression analysis of the release data. The best-fit model was the release model, exhibiting an R2 value approaching one.

### In vitro cell culture studies

#### In vitro cytotoxicity study

Cellular cytotoxicity was evaluated utilizing the MTT technique [33]. Experiments were performed according to the guidelines sanctioned by the Research Ethics Committee (IRB NO 00010556 - IORG 0008839) (0346_11/2021), Faculty of Dentistry, Alexandria University, Egypt. Cells (HSF or A-431) were implanted into 96-well plates (Corning, NY) at a density of 5 × 10^3^ cells per well in 100 µL of culture medium. Following a 24-h cell attachment period, the medium was replaced with treatments consisting of free AMG (10–80 mg/mL), blank nanocomposite (CS/rGO-ZnO/HA), and AMG-loaded nanocomposite (CS/rGO-ZnO/HA/AMG; equivalent to 1–640 µg/mL of AMG). After a 48-h incubation period, the treatment-containing media were aspirated, and 100 µL of fresh medium with 10 µL of MTT reagent (5 mg/mL) was added to each well. The plates were further incubated for an additional 4 h under light protection within a CO_2​_ incubator. The 48 h incubation period was deliberately selected based on the doubling time of A-431 cells, which is approximately 30 h. This interval ensures that the cells undergo at least one full division cycle, facilitating a distinct separation between proliferative and inhibited states influenced by the treatments. In addition, preliminary optimization studies indicated that 24 h was insufficient for the nanocomposite to exert its full cumulative cytotoxic effect or for the sustained release of AMG from the CS/rGO-ZnO/HA nanaocomposite to attain therapeutic levels. Conversely, a 72 h incubation interval was avoided to prevent over-confluency and spontaneous cell death in control groups due to nutrient depletion, which could yield false-positive results. Consequently, the 48 h timeframe offered an ideal opportunity to examine the therapeutic difference, while maintaining cellular viability in the control groups. [34] Next, DMSO (100 µL) was added to solubilize the formed formazan crystals. Absorbance was investigated at 570 nm by a microplate reader (Bio-Rad microplate reader). Cell viability was calculated as follows:

2 $$\%\mathrm{Viability}\;=\frac{\mathrm{Absorbance}\;\mathrm{of}\;\mathrm{sample}\;\mathrm{at}\;570\;\mathrm{nm}}{\mathrm{Absorbance}\;\mathrm{of}\;\mathrm{control}\;\mathrm{at}\;570\;\mathrm{nm}}\;\times\;100$$

Cells treated with culture medium only served as control cells (representing 100% viability). Cell viability was expressed as a percentage of inhibition relative to the sample concentration, which was used to determine the IC_50_ value. Data were analyzed using GraphPad Prism 7, and results were represented as mean ± SD. 

#### Wound scratch assay

The wound scratch assay was utilized to evaluate the potential of free AMG, blank nanocomposite, and AMG-loaded nanocomposite to inhibit cancerous cellular migration [35]. Cells were cultured in a 6-well plate for a duration of 24 h till reaching 70–80% confluency to ensure cellular adhesion. The formed cellular monolayer was scratched by a sterile yellow tip to form a wound within the cellular monolayer. Detached cells were taken off by washing twice with sterile PBS. The culture media were replaced with fresh media, containing either free AMG, AMG-loaded nanocomposite, or blank nanocomposite. To ensure a standardized comparative analysis, the concentration of each treatment was normalized to its respective half- maximal inhibitory concentration (0.5 IC_50_). The zero-time point images of the scratch were captured using an inverted phase-contrast microscope (Olympus, Massachusetts, USA). The cells were subsequently incubated with the treatments, and images were captured after 24 and 48 h. Control cells receiving no treatment were considered to have 100% migration, and the migration of the treated cells was determined as a ratio to control cells. Results from the three independent experiments were calculated as the mean ± SD, and representative images were analyzed to calculate the wound healing area using ImageJ software (Version 1.52).

The percentage of wound closure was calculated as follows:

3 $$\%Wound\;closure\;=\;\frac{Wound\;area\;at\;zero\;time-Wound\;area\;at\;specific\;time\;interval}{Wound\;area\;at\;zero\;time}\;\times\;100$$

#### In vitro cellular uptake

To evaluate the nanocomposite uptake by A-431 cell line, Rhodamine B was encapsulated within the nanocomposite matrix instead of the drug to be used as a fluorescent probe. Fresh aqueous Rhodamine B solution was prepared (0.01 mg/mL), then 20 µL of Rhodamine B solution was added dropwise to (CS/rGo-ZnO) mixture. The mixture was allowed to stir for 2 h. It was crosslinked with TPP to ensure incorporation of Rhodamine B within the nanocomposite matrix, and then the nanocomposite was coated with HA. Afterwards, the dye-loaded nanocomposite pellets were recovered by high-speed centrifugation for 10 min at 5200 xg, and washed three times with deionized water to remove free Rhodamine B [36]. A-431 cells were implanted in a 24-well plate at a concentration of 10^5^ cells/well, followed by incubation for 24 h at 5% CO_2_ and 37 °C until 80% confluence. Cells were then incubated with either free Rhodamine B solution or an amount of the nanocomposite containing 0.1 µg/mL of rhodamine B, followed by incubation in the dark for 24 h. Nanocomposites that were not internalized within the cells were removed via washing with PBS, pH 7.4, followed by fixation for 10 min with freshly prepared paraformaldehyde (4%, pH 7.4). Untreated cells were included as a negative control to quantify innate background fluorescence (autofluorescence). These values were subsequently used to calibrate the fluorescence intensity of the treated groups, ensuring that all obtained data were corrected for cellular and instrumental background noise. Finally, cells were mounted with DAPI mounting medium (VECTASHIELD, Vector Laboratories, Inc. Burlingame, CA) for nucleus staining, and stored at 4 °C under light protection till visualization. Cells were visualized at the excitation bands of rhodamine B (530–615 nm) and DAPI (358–416 nm) using a confocal laser scanning microscope (Leica DMi8, Lecia CMs-GmbH, Germany). The fluorescence intensity was determined by the corresponding Lecia software [37].

### Statistical analysis

Data were analyzed using IBM SPSS Statistics software (*Version 20.0*; IBM Corp., Armonk, NY, USA). The normality of continuous data was assessed using the Shapiro-Wilk test. Quantitative variables were expressed as mean ± standard deviation (SD) and range (minimum and maximum). For comparisons between two groups of normally distributed data, the unpaired Student’s t-test was employed. For multi-group comparisons, one-way analysis of variance (ANOVA) test was utilized, followed by Tukey‘s post hoc test for subsequent pairwise comparisons. Statistical significance was defined as a P-value ˂ 0.05 (5% significance level).

## Results

### Preparation and determination of EE% of amygdalin (AMG)-loaded (CS/rGO-ZnO/HA) nanocomposite

The data presented in Table 1 revealed that an increase in CS concentration correlates with a rise in EE%. Increasing the ratio of CS to HA from 6:1 to 10:1 results in a higher EE%. Regarding the drug amount factor, the data showed that the EE% increased with higher drug concentrations, at the same CS amount and CS: HA ratio.

### Physicochemical characterization

Table 1 shows the average hydrodynamic diameters (particle size) of the various AMG-loaded (CS/rGO-ZnO/HA) nanocomposites. The dynamic light scattering (DLS) analysis revealed that the particle size was significantly influenced by the concentration of the incorporated components, with all formulations maintaining a nanometric scale. Results have demonstrated that elevating CS concentration in the range from 0.25 to 0.5 g leads to the formation of smaller particles. While the utilization of a smaller amount of HA in the ratio of 10:1 leads to the formation of reduced particles compared to a 6:1 ratio. Increasing the drug concentration reduced the particle size (Table 1).

Results demonstrated in Table 1 revealed that ZP of all the prepared formulations were negatively charged and higher than − 20 mV, indicating good stability of the prepared NPs, which can prevent aggregation and promote dispersion of the NPs in the bloodstream. Based on the results obtained from the EE%, PS, and ZP, F6 was selected as the optimal formulation for further analysis. The optimal composition for the AMG-loaded (Cs/rGO-Zno/HA) NPs was identified as 500 mg CS, with an AMG loading of 15% relative to the CS weight (75 mg) and a CS: HA weight ratio of 10:1 (w/w). The specific formulation yielded superior physicochemical properties, characterized by a minimum hydrodynamic diameter of 180 ± 2.13 nm, high colloidal stability with a zeta potential of -36.5 ± 4.96 mV, and a maximum EE% of 90.81% ±1.23.

### Morphological examination

**Fig. 2:**
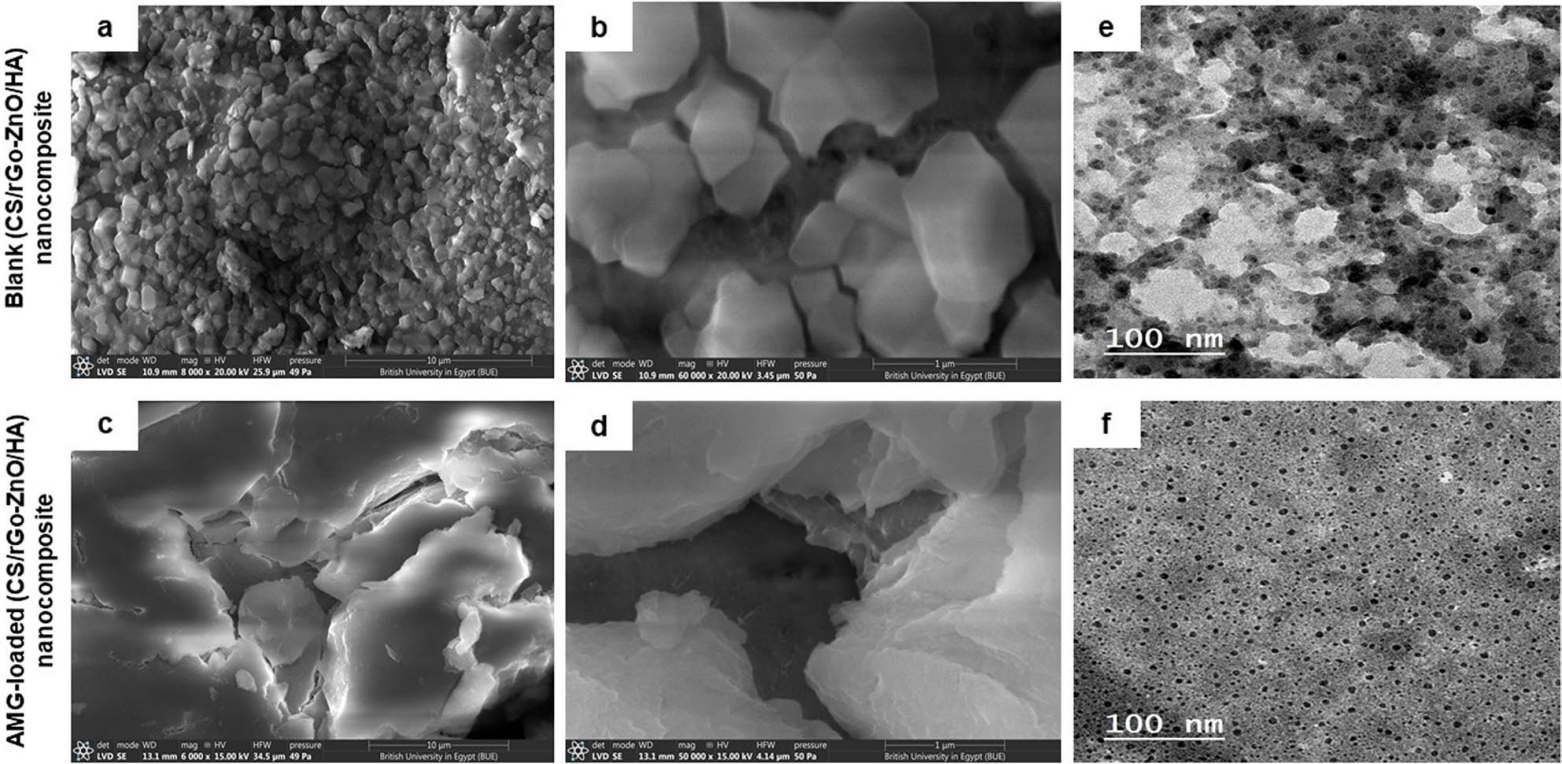
Morphological characterization of the nanocomposites. SEM images (surface view) of blank (CS/rGO-ZnO/HA) nanocomposite (**a**, **b**) (Original magnification 8000X and 60,000X, respectively.) and AMG-loaded (CS/rGO-ZnO/HA) nanocomposite (**c**, **d**) (Original magnification 6000X and 50,000X, respectively). SEM images of AMG-loaded nanocomposite revealed the maintenance of structural integrity post-loading. TEM images (cross-sectional view) of blank (CS/rGO-ZnO/HA) nanocomposite (**e**), and AMG-loaded (CS/rGO-ZnO/HA) nanocomposite (**f**). (Scale bar = 100 nm), confirming the internal distribution of spherical ZnO and rGO within the polymeric matrix

The morphology of the surface via SEM visualization revealed a smooth surface and good porosity owing to the strong hydrophilicity of HA, and its great potential to swell in water (Fig. 2a-d**)**, whereas TEM images showed homogenous dispersion of the spherical rGO and ZnO NPs within the nanocomposite (Fig. 2e and f**)**.

### Fourier transform infrared spectroscopy (FTIR) and X-ray diffraction (XRD)

**Fig. 3:**
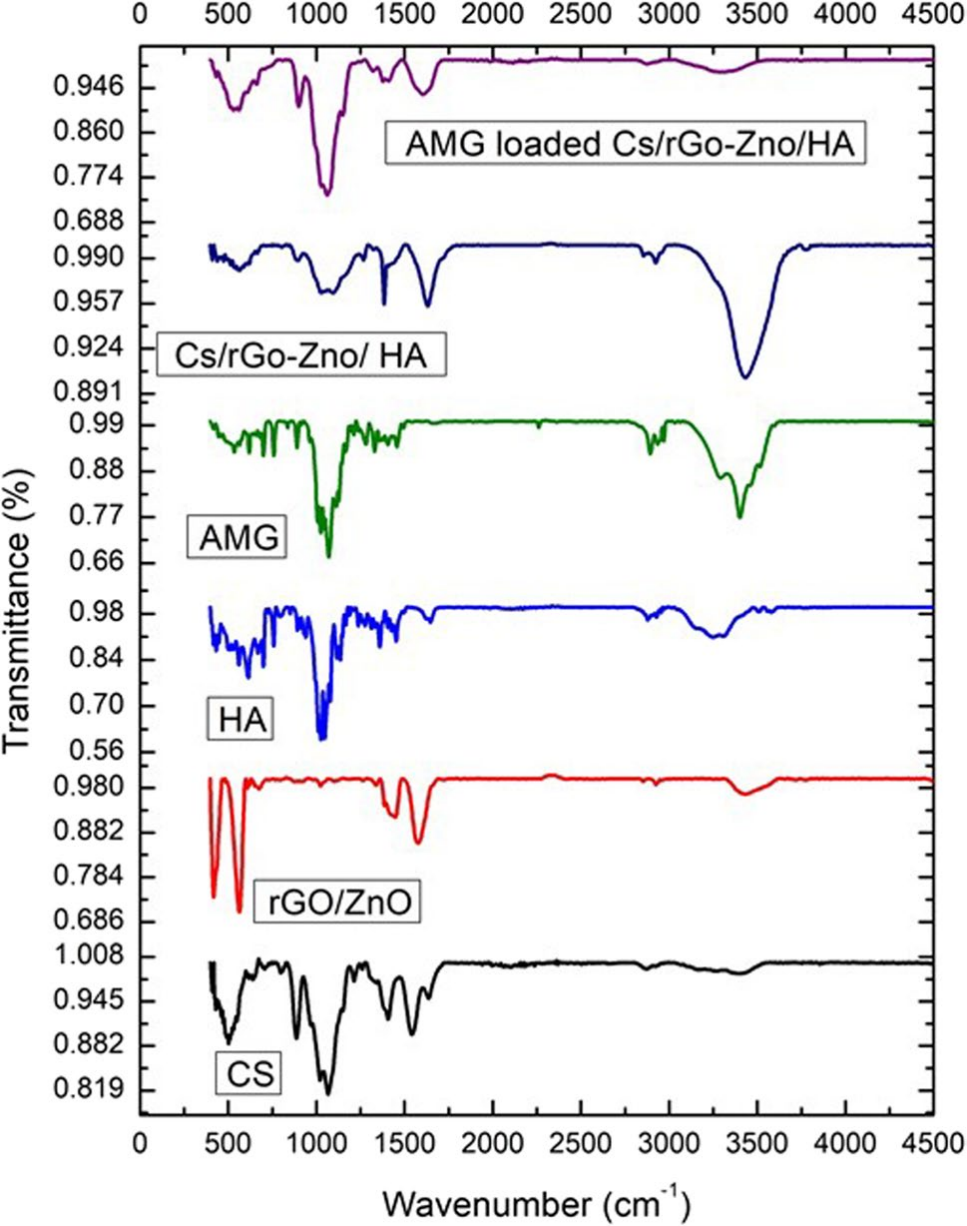
FTIR spectra of blank (CS/rGO-ZnO/HA) nanocomposite, AMG-loaded (CS/rGO-ZnO/HA) nanocomposite, and individual components

**Fig. 4:**
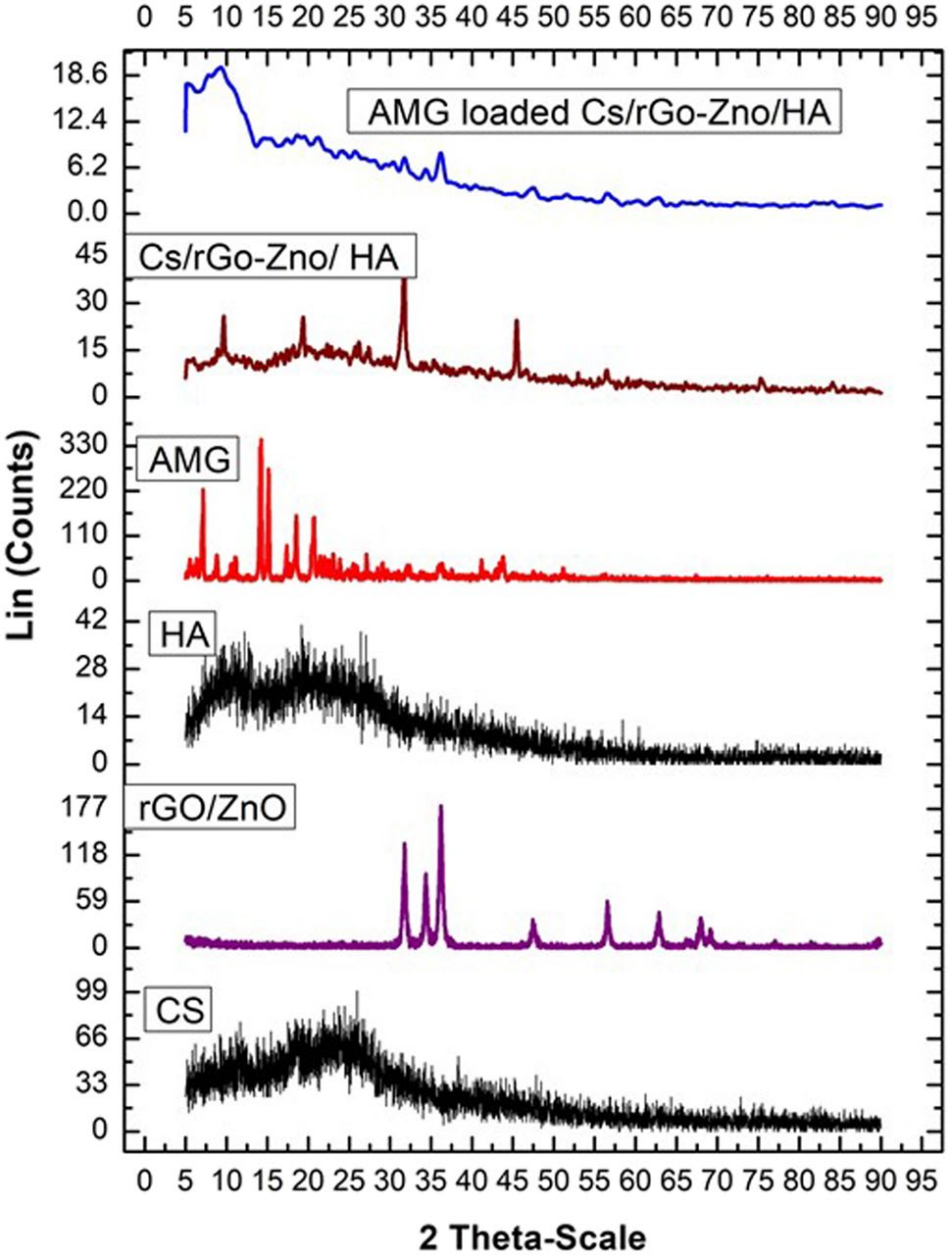
XRD patterns of the synthesized materials. Comparative diffractograms of individual components, blank CS/rGO-ZnO/HA nanocomposite, and AMG-loaded CS/rGO-ZnO/HA nanocomposite. Results confirmed successful integration of ZnO and rGO into the polymeric matrix and the amorphous state of encapsulated AMG within the quaternary nanocomposite system

Figure 4 clearly shows the IR spectra of individual components, including CS, rGO-ZnO, HA, and AMG, as well as drug-free (CS/rGO-ZnO/HA) nanocomposite and AMG-loaded (CS/rGO-ZnO/HA) nanocomposite. As shown in Fig. 3, the FT-IR spectrum of CS showed that the hydroxyl and amine groups were represented by a broad band at 3353 cm^− 1^, the weak peak observed at 3400 cm^− 1^ is attributed to the -NH2 and -OH groups stretching vibration and intermolecular hydrogen bonding [38], while the peak at 2867 cm^− 1^, the bending vibrations of N–H (N-acetylated residues, amide II band) at 1591 cm^− 1^, and the absorption band of the carbonyl group (C = O) stretching of the secondary amide (amide I band) at 1625 cm^− 1^ were all caused by –OH stretching [39]. The amide and ether bond N-H stretching and the amide II band N-H stretching are the causes of the peaks at 1450 and 1382 cm^− 1^, respectively. The two peaks seen at 1060 and 1022, respectively, were principal OH (typical peak of –CH2–OH in primary alcohol, C–O stretch) and secondary hydroxyl group (typical peak of CHOH in cyclic alcohols, C–O stretch) [40, 41]. While the illustrated FTIR spectrum of rGO/ZnO revealed the characteristic peaks of the rGO and ZnO at 1375 cm^–1^ and 558 cm^–1^, corresponding to the vibration modes of the C-O-H and Zn-O stretching, respectively [42, 43]. Furthermore, the FTIR spectrum of HA showed three prominent bands at 3300 cm^–1^, which can be related to hydrogen-bonded O-H and N-H stretching vibrations of the N-acetyl side chain [44], at 1600 cm^–1^, and 1415 cm^–1^, which can be assigned to the asymmetric (C = O) and symmetric (C-O) stretching modes of the planar carboxyl groups in the hyaluronate skeleton [45]. FTIR spectrum of AMG was distinguished by three characteristic peaks at 3400 cm^–1^, 2260 cm^–1^, and 1460 cm^–1^ corresponding to the vibrational modes of OH (stretching), C ≡ N (stretching), and aromatic C = C (stretching), respectively [46]. Illustrative IR spectrum of AMG-loaded (Cs/rGO-ZnO/HA) nanocomposite exhibited a shift in the OH/NH group peak to approximately 3477 cm^–1^, suggesting hydrogen bonding between the drug and the (Cs/rGO-ZnO/HA) nanocomposite. The spectra exhibited characteristic peaks at 2180 and 1465 cm^–1^, assigned to the C ≡ N (stretching) and aromatic C = C (stretching) of the drug.

Figure 4 demonstrated the results of XRD analysis. Results revealed that CS exhibits a very broad peak between 2θ = 10° and 2θ = 20° [47]. While the XRD spectrum of rGO-ZnO revealed a diffraction peak at 2θ = 31.4°, indicating the distance between graphene layers, and a diffraction peak at 2θ = 47.74°, indicating a short-range order in stacked graphene layers [48]. It also revealed sharp characteristic peaks at f 33.9, 35.5, 57.7, and 62.9, which are reported for the zinc oxide structure, confirming its crystallinity [49]. XRD analysis of HA presented broad peaks between 2θ of 10° and 20°, reflecting its amorphous nature [50]. Furthermore, AMG spectrum showed a characteristic sharp peak located in the low-angle region, exactly at 2 θ of 5.69. and sharp peaks at 2θ between 14° and 23°, which confirmed its crystalline nature [51]. The AMG-loaded (CS/rGO-ZnO/HA) nanocomposite spectrum displayed a shift with reduced intensities of certain peaks or their complete disappearance in comparison to the pure compounds.

### Differential scanning calorimetry (DSC) and thermogravimetric analysis (TGA)

The thermograms as well as the phase transition temperatures (T_c_) of individual components, including CS, HA, AMG, rGO-ZnO, as well as drug-free (CS/rGO-ZnO/HA) nanocomposite and AMG-loaded (CS/rGO-ZnO/HA) nanocomposite, are demonstrated in (Fig.S1). The DSC thermogram of CS showed an endothermic peak at 63.55 and 214.44 °C [52]. While the DSC thermogram of rGO-ZnO showed sharp exothermic peaks, which are related to ZnO at 383.08 and 544.15 °C [53] with no obvious range of melting temperatures for rGO as previously documented [54]. Furthermore, DSC of HA showed an exothermic sharp peak at 233.66 °C, which could be associated with its thermal decomposition [55, 56]. The DSC curve of AMG revealed sharp endothermic peaks at 223.44 °C [16].

The TGA thermograms of individual components, including CS, HA, AMG, rGO-ZnO, as well as drug-free (CS/rGO-ZnO/HA) nanocomposite and AMG-loaded (CS/rGO-ZnO/HA) nanocomposite, are demonstrated in (Fig.S2). According to the TGA thermograms of CS, there is a weight loss of approximately 72.27, which is characterized by initial weight loss in the range of 25 to 160 °C due to evaporation of the absorbed water [57], while the weight loss shown in the temperature range from 150 to 900 °C could be due to the breakdown and degradation of the polymer chain of CS, the breaking down of hydrogen bonds between free amino groups and the N-acetyl groups, the gradual degradation of carbonaceous residue, and the byproducts formed during the earlier stages [30, 58]. Furthermore, TGA for rGO/ZnO displayed a weight loss of approximately 10.67% which can be attributed to the removal of moisture content with good stability until 500 °C [59, 60]. The TGA thermogram of HA displayed a weight loss of approximately 85.32%. Its thermogram revealed two mass losses: an initial loss at 70–100 °C, due to possible evaporation of water [61], and a mass loss between 100 and 300 °C, which may be due to a partial degradation in the HA molecular structure [62]. TGA thermogram of AMG revealed a weight loss of about 96.98% of its initial weight, which is characterized by initial weight loss due to evaporation of water below 220 °C, followed by thermal decomposition after melting, which takes place in the temperature range between 246 and 481.6 °C [63].

### In vitro drug release study

**Fig. 5:**
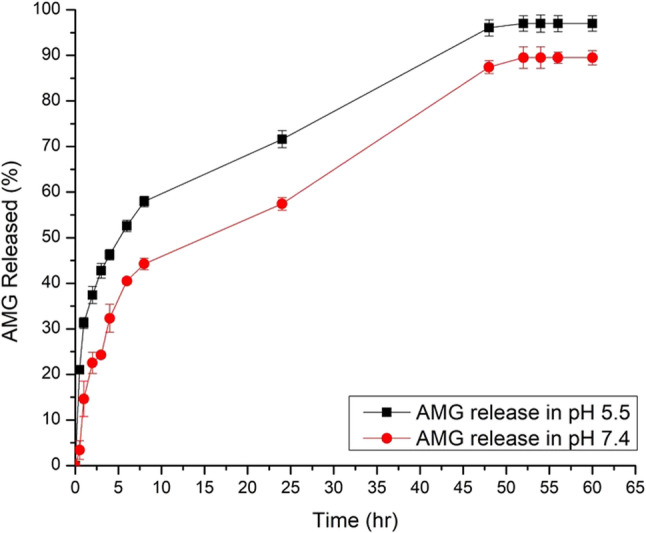
In vitro release profiles of AMG from (CS/rGO-ZnO/HA) nanocomposite in PBS (pH 5.5) and PBS (pH 7.4) release media. Comparative release profiles of AMG from the CS/rGO-ZnO/HA nanocomposite in simulated physiological (pH 7.4), and acidic tumor microenvironment (pH 5.5) conditions at 37 °C. Results are expressed as mean ± SD (*n* = 3)

The release profile of AMG from AMG-loaded (CS/rGO-ZnO/HA) nanocomposite at 37 °C ± 0.5 at pH 5.5 and pH 7.4 is shown in Fig. 5. Results showed that AMG release from AMG-loaded CS/ /rGO-ZnO /HA nanocomposite was slower in alkaline medium compared to the acidic medium. The results show that a continuous and controlled AMG release occurred over 52 h for both acidic and alkaline media. The % of AMG released after 8 h (Q_8h_) from AMG-loaded (CS/rGO-ZnO/HA) nanocomposite in acidic medium and alkaline medium were 57.93 ± 1.45% and 44.25 ± 1.25%, respectively. Statistical analysis of Q8h revealed that the release of AMG from the developed NPs exhibited significantly higher values in acidic medium than its corresponding in alkaline medium (*p* < 0.05).

At acidic pH, there was a notable initial burst release within the first 4 h, accounting for about 66% of the total drug. The remaining 30% of the drug was then released in a sustained profile over 52 h. This suggests that the ionically crosslinked CS NPs were highly effective in encapsulating a large quantity of AMG owing to the electrostatic attraction between the positively charged CS and the negatively charged AMG. This efficient encapsulation likely contributed to the notable difference in drug release profiles observed between pH 5.0 and pH 7.4. Figure 5 also revealed that AMG release from the developed nanocomposite exhibited a biphasic release profile in both media. A fast release phase results from the initial release of AMG adsorbed onto the surface of the developed NPs, causing a rapid release after dispersion into the release medium. Table 2 showed the results of analyzing release profiles using multiple kinetic models. The release pattern of AMG from the developed NPs in both media was found to meet the Higuchi model (diffusion-regulated mechanism), as evidenced by the highest correlation coefficient (R^2^) values.

**Table 2:** The calculated correlation coefficients and kinetics parameters of in vitro AMG release profile from the developed AMG-loaded (CS/rGo-ZnO/HA) nanocomposite in neutral and acidic pH

Release medium	Q_8h_ ± SD (%)	Zero order	First order	Higuchi	Peppas	
		*R* ^2^			*R* ^2^	*N*
PBS (pH5.5)	57.93 ± 1.45	0.9153	0.7615	0.9785	0.9439	
PBS (pH7.4)	44.25 ± 1.25	0.9119	0.5550	0.9734	0.8023	

### In vitro cell culture studies

#### In vitro cytotoxicity study

**Fig. 6:**
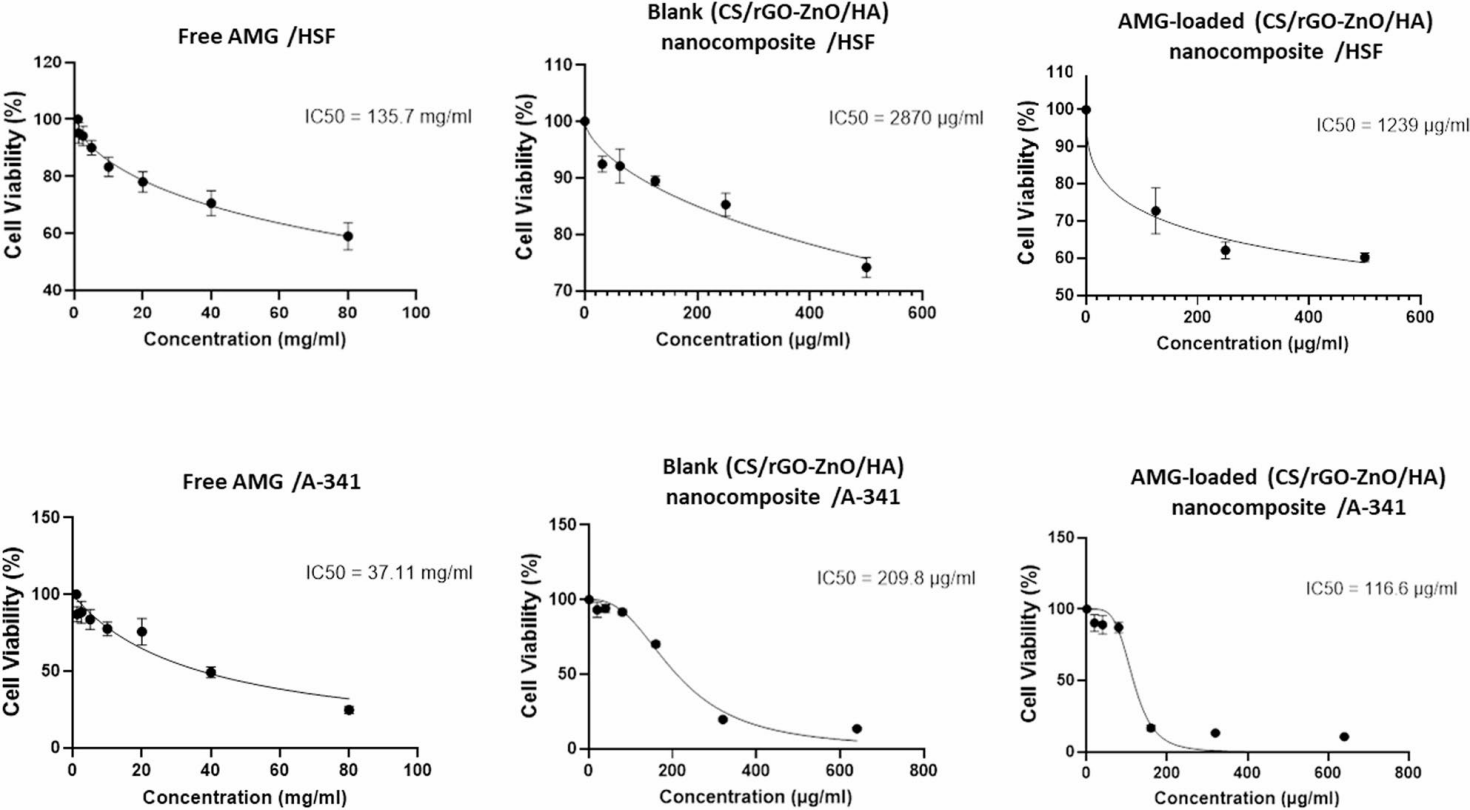
In vitro cytotoxicity of free AMG, blank (CS/rGO-ZnO/HA) nanocomposite, AMG-loaded (CS/rGO-ZnO/HA) nanocomposite on human fibroblast (HSF) cells and human squamous cell carcinoma **(**A-431) cells after incubation for 48 h. Cell viability test was assessed by using MTT assay

As shown in Fig. 6; Table 3, free AMG exhibited very low or even negligible cytotoxic effect on HSF with an IC_50_ of 135.7 ± 12.2 mg/mL, whereas blank nanocomposite and AMG-loaded nanocomposite exhibited IC_50_ values of 2870 ± 229.6 µg/mL and 1239 ± 86.7 µg/mL, respectively. Blank nanocomposite and AMG-loaded nanocomposite showed relatively low cytotoxic effects on HSF, confirming their good cytocomptability with normal cells.

**Table 3:** Comparison between IC_50_ values of different formulations in Human skin fibroblasts (HSF) and Human epidermoid squamous cell carcinoma (A–431) cell lines using MTT assay

Cell line	Mean IC_50_			
	Free AMG (*n* = 3)	Blank nanocomposite (*n* = 3)	AMG-loaded nanocomposite (*n* = 3)	*p*
Human skin fibroblasts (HSF)	135.7 ± 12.20 mg/mL	2870.0^$^ ± 229.6 µg/mL	1239.0^$@^ ± 86.7 µg/mL	< 0.001^*^
Human epidermoid squamous cell carcinoma (A–431)	37.11 ± 2.96 mg/mL	209.8^$^ ± 16.78 µg/mL	116.6^$@^ ± 10.49 µg/mL	< 0.001^*^

Data were expressed as mean ± SD.F: F for One way ANOVA test, Pairwise comparison between each 2 groups was done using Post Hoc Test (Tukey). p: p value for comparing between the studied groups. *: Statistically significant at *p* ≤ 0.05, **$**: Significant with Free AMG, **@**: Significant with Blank nanocomposite

**Fig. 7:**
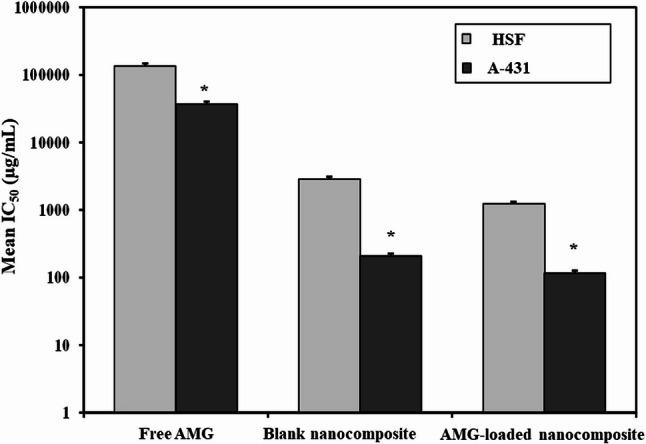
Comparative analysis of half-maximal inhibitory concentrations IC_50_ values of free AMG, blank CS/rGO-ZnO/HA, and AMG-loaded nanocomposites against HSF and A-431 cell lines. AMG-loaded nanocomposite exhibited a significant reduction in IC_50_ for A-431 cells compared to the free drug, indicating potentiated anticancer efficacy. The high IC_50_ values in case of HSF cells demonstrated the selective biocompatibility of the nanoplatform. Data are expressed as mean ± SD. *: Statistically significant at *p* ≤ 0.05

On the other hand, when the cytotoxic effect of free AMG was examined against A-431 cell line, it displayed an IC_50_ of about 37.11 ± 2.96 mg/mL, which was about 3.6 times lower than that observed in the case of HSF cell line (Fig. 7). Results revealed that blank nanocomposite (CS/rGO-ZnO/HA) and AMG-loaded nanocomposite displayed IC_50_ values of 2870 ± 229.6 µg/mL and 1239 ± 86.7 µg/mL, respectively, in the case of HSF cell line. In contrast, they displayed IC_50_ values of 209.8 ± 16.78 µg/mL and 116.6 ± 10.49 µg/mL, respectively, in the case of A-431 cell line. These results indicated that the cytotoxic effect of both blank nanocomposite and AMG-loaded nanocomposite was much more pronounced in cancer cell line (A-431) than normal cell line (HSF). AMG-loaded nanocomposite demonstrated the highest cytotoxic effect on A-431 cancer cells compared to all other examined formulations, with an IC_50_ of 116.6 ± 10.49 µg/mL, which was markedly lower than that of free AMG (37.11 ± 2.96 mg/mL) (Fig. 7).

#### Wound scratch assay

**Fig. 8:**
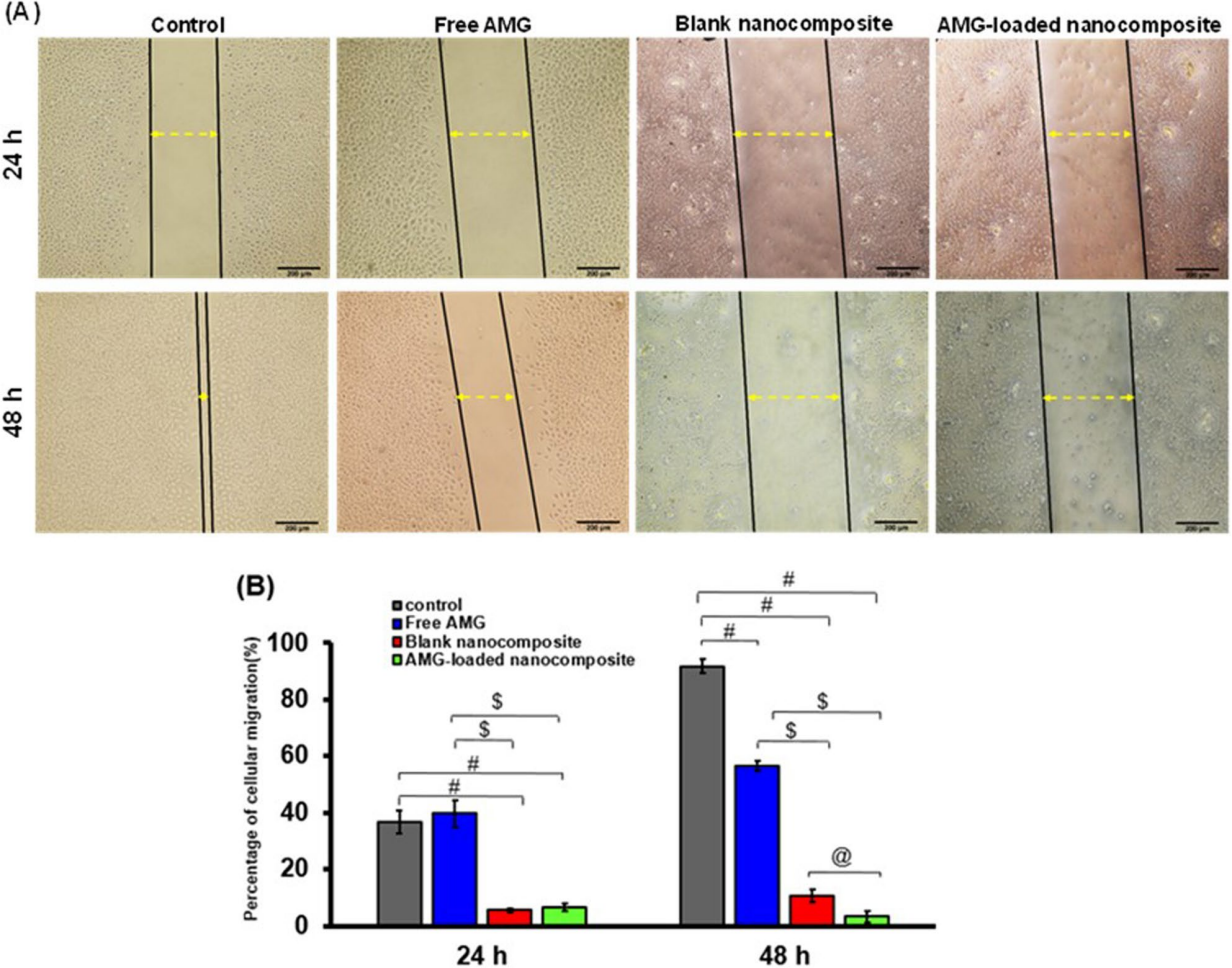
In vitro wound scratch assay of AMG-loaded nanocomposites on A-431 cells. Representative micrographs showing inhibitory effect of Free AMG, blank nanocomposite, and AMG-loaded nanocomposite on A-431 cell migration at 24 and 48 h time intervals (Scale bar = 200 μm) (**A**), and quantitative data analysis of the migration% (**B**). Data were represented as mean ± SD of 3 measurements. F: F for One way ANOVA test, Pairwise comparison between each 2 groups was done using Post Hoc Test (Tukey). p: p value for comparing between the studied groups.*: Statistically significant at *p* ≤ 0.05. #: Significant with Control, $: Significant with Free AMG, @: Significant with Blank nanocomposite

As demonstrated in Fig. 8, free AMG exhibited cellular migration of about 39.67 ± 4.64% after 24 h, whereas it displayed cellular migration of about 56.42 ± 1.89% after 48 h. On the other hand, blank nanocomposite exhibited cellular migration of about 5.49 ± 0.80% after 24 h and 10.50 ± 2.19% after 48 h, indicating that the blank nanocomposite displayed also an anti-migratory effect. AMG-loaded nanocomposite exhibited cellular migration of about 6.54 ± 1.39% and 3.22 ± 2.06% after 24 h and 48 h, respectively, indicating that the combination of AMG with the nanocomposite components significantly boosted the anti-migratory effect. This improved effect after 48 h might be due to the sustained release of AMG from the nanocomposite, which was able to provide a controlled maximum in vitro drug release after 48 h, and hence mitigating the cascading dynamic and time-dependent processes of cellular migration.

#### In vitro cellular uptake study

**Fig. 9:**
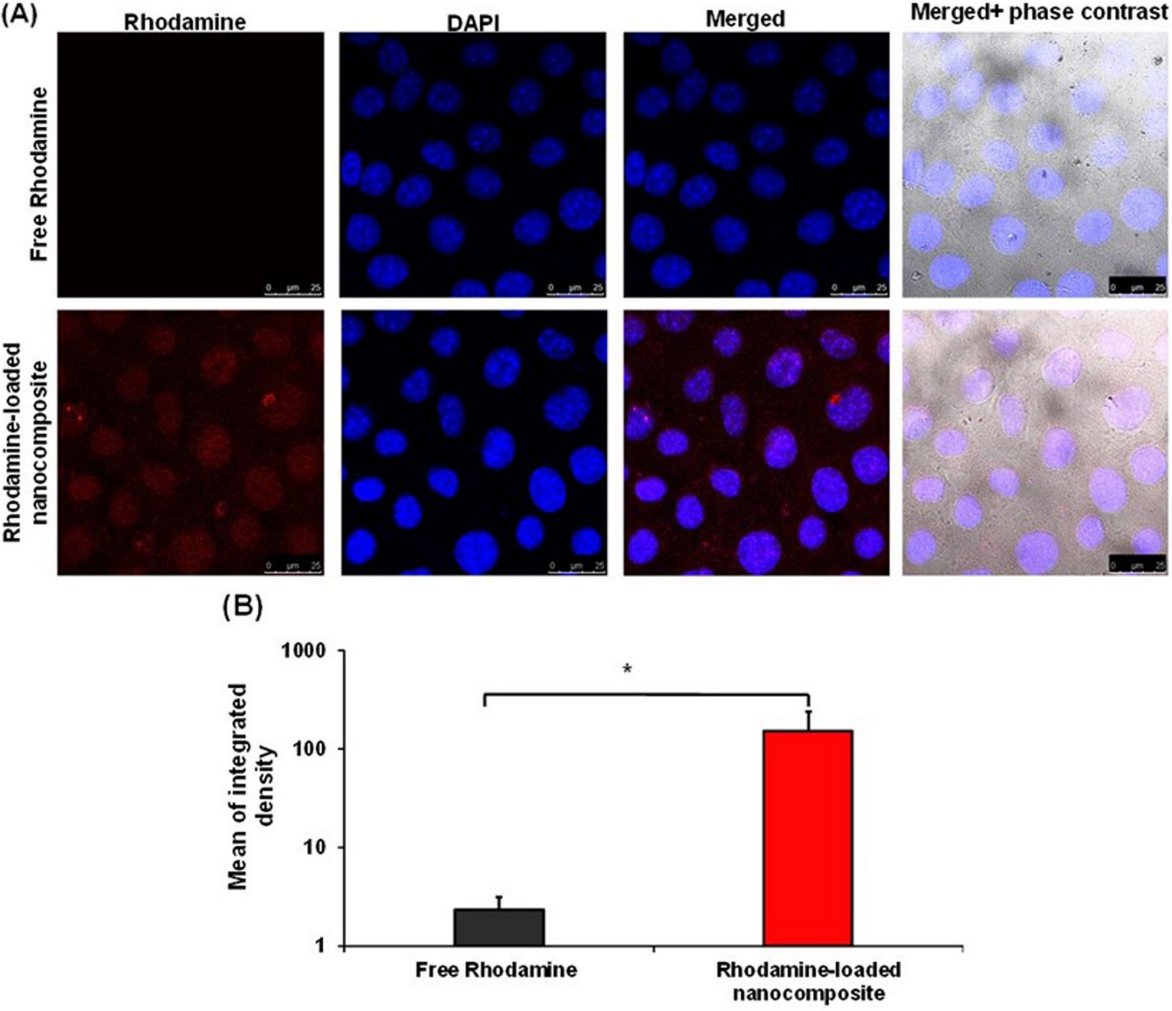
Comparative cellular uptake analysis via confocal laser scanning microscopy (CLSM). Representative fluorescence images of A-431 cells following 24 h of incubation at 37 °C with free Rhodamine B and Rhodamine B-loaded nanocomposites, demonstrating enhanced intracellular accumulation of the dye when encapsulated within the (CS/rGO-ZnO/HA) matrix (Scale bar = 25 μm) (**A**), and quantitative fluorescence intensity. *: Statistically significant at *p* ≤ 0.05 (**B**)

As shown in Fig. 9, encapsulation of rhodamine B within the prepared nanocomposite resulted in improved cellular internalization when compared to free rhodamine B. This improved cellular uptake could be due to the composition of the nanocomposite.

## Discussion

The present study describes the development and characterization of a novel quaternary nanocomposite designed for the encapsulation of amygdalin (AMG), a naturally occurring antineoplastic agent. The primary objective was to enhance its therapeutic efficacy while minimizing the dose-dependent systemic toxicity typically associated with its free form. Chitosan (CS) and hyaluronic acid (HA) complexes were utilized as the primary delivery matrix due to their well -documented biocompatibility, inherent mucoadhesive properties, and targeting delivery potential [17, 19, 64]. Furthermore, reduced graphene oxide doped with zinc oxide NPs (rGO-ZnO) was incorporated into the system to leverage its physicochemical properties, such as poor solubility and non-specific distribution [65, 66]. By integrating these four materials, we aimed to develop a nanoparticulate system capable of the targeted and sustained delivery of amygdalin.

The ion gelation method was used to successfully prepare AMG-loaded nanocomposite. This method depends on using CS or its derivatives with oppositely charged macromolecules or with a cross-linking agent such as tripolyphosphate (TPP) to form gels through ionic linkage [67]. Previous studies have mentioned that the higher CS concentration can make the surface matrix denser with a greater compact structure, causing a retardation in both the swelling and degradation of NPs and retention of the encapsulated drugs in the NPs [68, 69]. The ratio of hyaluronic acid (HA) in formulation can significantly impact the entrapment efficiency of active ingredients within drug delivery systems such as nanoparticles. Generally, increasing the HA concentration can enhance entrapment efficiency, especially with hydrophilic drugs such as AMG. However, this relationship is not always linear, and excessive HA can sometimes lead to decreased entrapment efficiency [70].

It has been documented that CS based NPs could encapsulate both water-insoluble and water-soluble drugs [71, 72]. Water-soluble drugs such as AMG are mixed with CS solution before NPs preparation, so drug loading onto CS NPs occurs during NPs formation by incorporation in the CS matrix, leading to physical interaction and high EE%, as observed in previous studies of AMG encapsulation in chitosan-alginate particles, in which the drug was entrapped into the NPs matrix [11].On the other hand, when CS concentration was increased, smaller PS was observed due to robust intramolecular electrostatic interactions, which facilitated the formation of small particle sizes. After adsorption, the significant attraction between segments of polyelectrolyte chains and oppositely charged surface groups of chitosan and amygdalin leads to their proximity, resulting in the fabrication of NPs with reduced size [73]. Additionally, increasing the drug concentration during preparation caused a reduction in the size because increasing the amount of drug encapsulated within the nanoparticles raises the negative charge, attracting more chitosan to the surface. This increased the negative charge on the surface, leading to a subsequent improvement in the stability of the NPs [74].

Drug loading has a significant effect on the physical parameters of (Cs/rGO-ZnO/HA) NPs, including particle size and zeta potential. Particle size is critical for vital processes such as cellular uptake, intracellular transport, and exocytosis, which affect the transepithelial transporting properties [75]. Ideally, stable CS based NPs can be in the range from 100 to 200 nm, with procedures such as homogenization, sonication, and nanoprecipitation, which can be used to manage size [76]. This range is ideal for passive targeting via the enhanced permeability retention (EPR) effect, providing a balance that allows for prolonged circulation periods, efficient extravasation through leaky tumor vasculature, and sufficient tumor tissue penetration [77]. .Moreover, a PDI less than 0.5 means a narrow distribution of the dispersed NPs, low interfacial tension, homogeneity, and fewer tendencies for aggregation [78].

Zeta potential is considered an important indicator of dispersion stability. The magnitude of the ZP indicates the amount of the electrostatic interactions between the dispersed NPs having the same charge. At low ZP levels (≤ 20 mV), attractive forces can overcome repulsion, resulting in aggregation. Dispersion enhances stability and prevents aggregation at a high ZP (≥ 20 mV) [79, 80].This stability is essential for drug delivery, as it ensures that nanoparticles can reach their target site without prematurely aggregating or being removed from circulation [81]. Additionally, the reticuloendothelial system (RES), primarily relying on macrophages in the liver and spleen, can clear NPs from the bloodstream rapidly. Negatively charged NPs are less likely to be recognized and removed by the RES, allowing them to circulate for longer periods and reach target tissues.

Furthermore, there was an inverse relationship between chitosan (CS) concentration and the magnitude of the negative zeta potential (ZP). Formulations synthesized with lower CS content exhibited more pronounced negative ZP values compared to those with higher CS concentrations. This phenomenon may be attributed to the polycationic nature of CS, where the protonated amino groups neutralize the negative surface charges of hyaluronic acid (HA) and tripolyphosphate (TPP) [82]. As the CS concentration increases, its positive charge density becomes more dominant, leading to a reduction in the overall net negative charge of the nanoparticles. Conversely, at lower CS levels, the anionic influence of TPP remains significant, resulting in higher negative ZP values [83].

FTIR was used to study the interaction between AMG and the NPs components. The results of IR-spectrum of AMG-loaded nanocomposite confirmed the successful loading of the AMG in (Cs/rGO-ZnO/HA) nanocomposite, as it was observed as a shift in the OH/NH group peak to about 3477 cm^–1,^ indicating the formation of hydrogen bonding between the drug and (Cs/rGO-ZnO/HA) nanocomposite. Furthermore, peaks at 2180 and 1465 cm^–1^, might correspond to the C ≡ N (stretching) and aromatic C = C (stretching) of the drug. The physical status of the developed NPs and the individual components was investigated via monitoring their crystallinity by XRD analysis. The spectrum of the AMG-loaded nanocomposite (CS/rGO-ZnO/HA/AMG) revealed that some peaks corresponding to the individual components had shifted to lower intensities, associated with the disappearance of certain peaks, which reflected the interaction between the different nanocomposite components and the drug [84].

Differential scanning calorimetry (DSC) is a widely used technique to determine phase transition temperatures, excess enthalpy, and interaction between components. The DSC was utilized to demonstrate the effect of the developed NPs on different components by studying their thermodynamic properties. Analysis of AMG-loaded nanocomposite thermal behavior displayed a shifting in the characteristic endothermic peaks of CS and a disappearance of the distinguished sharp peak of AMG. The high solubility and EE% of AMG in the developed AMG-loaded nanocomposite could explain the absence of its thermal peak [16]. TGA is used for studying the heat stability and weight variations of materials [57] . Analysis of AMG-loaded nanocomposite thermal behavior showed weight loss of about 56.45% characterized by initial weight loss at 179.44 °C due to evaporation of water. The difference in weight loss between free AMG and AMG-loaded nanocomposite (CS/rGO-ZnO/HA/AMG) confirmed the presence of AMG in the developed nanocomposite improved its thermal stability [85].

The drug release characteristics of chitosan-based nanoparticles were studied in vitro at different pH levels, simulating both normal physiological conditions (pH 7.4) and the acidic tumor microenvironment (pH 5.0). These experiments were conducted at 37 °C over 52 h. This focus on pH-responsive release is crucial because the lower pH found in tumor microenvironments, compared to the bloodstream, can enable targeted drug release. Such pH-sensitive delivery can potentially improve the therapeutic efficacy against solid tumors while simultaneously reducing adverse effects on healthy cells [86]. Release of AMG from the developed NPs exhibited significantly higher values in acidic medium when compared to physiological medium (*p* < 0.05), suggesting that AMG release from the developed nanocomposite was pH sensitive [17], with an increase in the amount of the released AMG as the pH was acidic. At lower pH, the amino (-NH2) groups within the chitosan structure undergo protonation, gaining a positive charge (NH_3_^+^). This protonation significantly increases chitosan’s solubility in water. This enhanced solubility, in turn, facilitates the release of encapsulated drugs, which are typically entrapped within the chitosan matrix. It is essential to note that this protonation process is reversible, meaning the degree of CS’s solubility and thus drug release is directly influenced by the surrounding pH. Conversely, at higher pH values, CS’s amino groups remain largely unprotonated and electrically neutral. In this state, chitosan adopts a gel-like structure, which effectively restricts the release of encapsulated drugs, thereby facilitating sustained release [87].

The observed biphasic release was due to the initial fast release of AMG adsorbed onto the surface of the developed NPs, causing a rapid release after dispersion into the release medium. This was subsequently followed by a sustained release, lasting for 52 h, due to diffusion of AMG from the interior of the NPs into solution via the tortuous pathway [88]. Nanocomposite prepared using CS could act as a drug depot, releasing the entrapped drug in a sustained manner. 

The cytotoxicity of free AMG, blank nanocomposite (CS/rGO-ZnO/HA), and AMG-loaded nanocomposite was examined on normal human fibroblast (HSF) and human squamous cell carcinoma (A-431) cell lines at various concentrations for 48 h by the calorimetric standard MTT assay. Interestingly, free AMG exhibited negligible cytotoxic effect on HSF. This was in consonance with previously reported research [89]. Moreover, some studies reported that free AMG can even have a protective effect on normal cells, as it was shown to minimize the apoptosis of normal breast and fibroblastic cells during chemotherapy. Moreover, 10 mM concentration of free AMG was found to exert cytoprotective effect on mesenchymal stem cell (MSC) culture exposed to staurosporine; an apoptosis inducer [90]. Several studies indicated that nanocomposites, including chitosan, reduced graphene oxide, and/or hyaluronic acid, displayed adequate cytocomptability with normal cell lines (NIH/3T3-L1, L929, or human fibroblasts) [91–93].

When free AMG was examined against A-431 cancer cell line, it exhibited lower IC_50_ in comparison to normal HSF cell line, which was in accordance with previous studies which reported that free AMG extracted from apricot seeds and almonds exhibited an IC_50_ of 100 µg/mL and 50 µg/mL, respectively, when examined against the human oral cancer cell line [94]. Moreover, the anticancer efficacy of AMG is highly selective towards cancer cells. Cancer cells are well-known to be overexpressing β-glucosidase (100–3600% higher than that of normal cells), a key molecule in the cytotoxic effect of AMG. This enzyme can hydrolyze AMG, producing hydrocyanic acid and glucose [8, 95]. The released cyanide ion can attack the mitochondrial cytochrome C oxidase, causing an inhibition of cellular respiration [96]. In addition, cyanide ions can overproduce reactive oxygen species (ROS), leading to an elevation in benzaldehyde production, which is associated with direct protein oxidation and lipid peroxidation [97]. Consequently, it seemed that AMG’s lethal effect on cancer cells is mainly due to the synergistic action of the two main products, hydrocyanic acid and benzaldehyde [12].

Conversely, normal cells possess the detoxifying mitochondrial enzyme ‘‘rhodanese’’, which can convert hydrogen cyanide and benzaldehyde into non-toxic thiocyanate (rhodanide) and benzoic acid, respectively [98]. The produced thiocyanate was found to be about 200 times less toxic compared to cyanide. Fortunately, cancer cells completely lack the detoxifying enzyme rhodanese, which prevents AMG from being converted to non-toxic metabolites. These unique features in cancer cells lead to overproduction and accumulation of toxic hydrocyanic acid and benzaldehyde metabolites, which cannot be detoxified due to a deficiency in rhodanese enzyme [98].

The divergent metabolic pathways through which malignant and normal cells process AMG may account for the significantly lower *IC*_*50*_ values observed in the A-431 cell line compared to the HSF cell line, suggesting that AMG, even in its free form, can serve as a targeting anticancer therapy. However, the AMG’s therapeutic potential can be further maximized by its encapsulation within a multi-component (CS/rGO-ZnO/HA) nanocomposite matrix. This strategic integration was designed to overcome pharmacological limitations of the free drug by providing a synergistic delivery platform that enhances stability and facilitates controlled release at the target site.

Nanoplatforms incorporating chitosan and reduced graphene oxide nanoparticles were cytotoxic on human lung cancer cells (A549), while they were less toxic on normal human fibroblasts (HFF cells) [99]. In another study, a nanocomposite comprised of (rGO-ZnO/ HA) was examined against the breast cancer cell line, giving an IC_50_ of 200 µg/mL, which is closely aligned with the results reported in this study [100]. Furthermore, rGO nanoparticles alone were proven to be highly toxic on the human breast cancer cell line (MCF-7), while exhibiting very low cytotoxicity on human normal breast cell line (MCF-10), which might indicate a selective cytotoxicity mechanism for the nanocomposite components [101]. In addition, the anticancer efficacy of rGO is often dose-dependent and may be enhanced through combination with other anticancer treatments [102]. A combination of rGO and ZnO nanoparticles in nanocomposites demonstrated superior antitumor efficacy compared to each individual component. The enhanced effect is believed to result from elevated ROS production in cancer cells, leading to mitochondrial malfunction and disruption of several cellular mechanisms [103].

Hyaluronic acid (HA) serves as a natural ligand for CD44 receptors, commonly overexpressed in malignant cells, thereby facilitating the active targeting of the nanocomposites towards cancer cells [104]. The significant cytotoxicity observed by the blank carrier in A-431 may be attributed to the intrinsic antineoplastic properties of its constituents. Furthermore, the observed potent therapeutic efficacy of AMG-loaded nanocomposite against cancerous cells likely arises from a synergistic mechanism involving pH-responsive release from chitosan and HA- mediated internalization, complemented by the cytotoxic effects of rGO and ZnO NPs [102, 105]. Notably, the integration of Zinc potentially boosts the efficacy of AMG, Consistent with previous reports of enhanced anticancer activity in metal -drug combinations [22, 23].

While the blank nanocomposite exhibited an augmented cytotoxic effect against A-431 cancer cells, the integration of AMG into the nanocomposite resulted in an IC50 threshold at a significantly lower concentration than the blank carrier, as the sensitivity of the blank carrier is primarily driven by oxidative stress (ROS) generated from rGO and ZnO NPs. Nonetheless, cancer cells activate survival pathways (such as Nrf2 signaling) to adapt to ROS. However, once amygdalin is better internalized within cancer cells via HA-mediated receptor targeting [104], and begins to hydrolyze into hydrocyanide, it inhibits cytochrome C oxidase in mitochondria, resulting in a secondary biochemical attack that drives the compromised cell towards irreversible apoptosis (cytotoxic) rather than a temporary growth arrest (cytostatic ). [104]. Furthermore, the synergistic combination of amygdalin within a nanocomposite permits a reduced total dosage to be administered, thereby minimizing the long-term accumulation of rGO and ZnO in healthy tissues, which enhances the overall safety profile. Normal cells such as HSF have a robust antioxidant defense system that can neutralize these produced ROS, and hence reduce their destructive effect on the cells [106]. It seemed that a synergistic nanocomposite comprised of CS, rGO, ZnO NPs, and HA will indeed improve the efficacy of the loaded drug due to its integrated complementary effect.

A wound healing or scratch assay is usually utilized to quantify cellular migration across a gap induced by in vitro scratching. This assay is frequently employed in oncology research to provide a rapid indication of whether specific medications can influence or inhibit cancer metastasis or proliferation [35]. AMG can inhibit cancer cells’ migration via modulating some cell adhesion molecules (integrins) [107] minimizing adhesion to the ECM and inhibiting invasion via matrix metalloproteinases (MMPs) [108].These effects are frequently coupled with its broader anticancer actions, like inducing apoptosis and cell cycle arrest [7]. However, some studies showed that AMG can have a stimulating effect on cellular proliferation and migration, indicating that the effect of AMG can vary according to the type of cells [107, 109]. This was consistent with our results, which showed that free AMG did not exhibit any inhibition on the migration of A-431 squamous cancer cells, which might be attributed to the possible development of resistance in the functional switch of some integrin receptors, leading to an enhancement in tumor cell migration [110].

Chitosan can be considered a direct anticancer agent as it can inhibit cancer cells’ migration via multiple mechanisms. Among these, its positive charge can strongly interact with the negatively charged components of cancer cell membranes, causing alterations in cell-cell and cell-matrix adhesion, which are essential for cell migration and invasion [111, 112]. In addition, chitosan was found to decrease the expression and activity of MMP-2 and MMP-9, which are enzymes responsible for breaking down the ECM, aiding in cancer cell invasion and metastasis [113].

The role of hyaluronic acid in cancer proliferation and metastasis often depends on its molecular weight. High-MW. HA mainly exerts an inhibitory or homeostatic effect on cancer cell migration, unlike low-MW. HA. [64]. High-MW. HA is usually related to the suppression of cell proliferation and migration. Moreover, it can act as a physical barrier, forming a dense matrix that might hinder cellular movement, and hence hindering migration [114]. In addition, high-MW. HA can also block pro-migratory or pro-oncogenic signaling pathways by binding to HA receptors like CD44 or RHAMM [19]. Regarding rGO, several studies revealed that it can inhibit in vitro proliferation and migration of multiple cancer cell lines, including cervical, prostate, breast, and neuroblastoma cancers [65]. rGO can inhibit cellular migration via various mechanisms, including ROS overproduction [65], cytoskeletal disruption [115], and stimulating apoptosis and cell cycle arrest [101]. Similarly, ZnO NPs were reported to induce cancer death via inducing S-phase arrest, and hence reducing colony formation and metastasis [116].

Incorporation of AMG into the developed nanocomposite showed the most potent anti-migratory effect compared to the blank carrier. These results were in accordance with previously published work, which showed that encapsulation of AMG into β-cyclodextrin NPs showed a potent anticancer effect on MCF-7 human breast cancer cells when compared to free AMG [117].Indeed, the synergistic effect of combining AMG with chitosan, rGO, ZnO NPs, and high-MW. HA showed great potential for significant anti-migratory effects. This boosted effect could be attributed to the multifunctional properties of the nanocomposite, which can hit multiple targets all at once. This multifunctional design of a nanocomposite could afford an advanced strategy in fighting cancer.

Cellular uptake assay is used to examine the mechanism by which a formulated therapeutic agent can internalize and accumulate in cancer cells [118]. The prepared nanocomposite enhanced cellular internalization through two key mechanisms: passive and active targeting. The passive targeting is achieved primarily through the Enhanced Permeability and Retention (EPR) effect. The nanocomposite’s optimized size, typically in the range of 10–200 nm, allowed it to extravasate from the leaky vasculature characteristic of solid tumors and accumulate in the tumor interstitium. The impaired lymphatic drainage in the tumor microenvironment (TME) further hindered the rapid clearance of the nanocomposite, resulting in its prolonged retention and localized accumulation, which represents a significant improvement over small-molecule drugs [77]. The pH-responsiveness of the chitosan component, which can change its conformation in the acidic environment of tumors, causes the release of its payload in the acidic TME [86].

Furthermore, the cationic nature of chitosan can enhance the uptake of the nanocomposite by macrophages compared to neutral particles, highlighting the importance of surface charges in phagocytosis and cellular entry [119]. In addition, surface modification or grafting of chitosan NPs with other materials, such as rGO/ZnO and/or HA, can “stealth” the NPs, leading to prolonged circulation time via reducing their recognition and clearance by the reticuloendothelial system (RES) [120].

HA-grafted chitosan nanocomposites can specifically target CD44 receptors, a key marker for squamous carcinoma cancer stem cells (CSCs). It is not just a passive marker but an active participant in key processes like self-renewal, invasion, and resistance to therapy, making it a promising target [121]. This “lock-and-key” mechanism can facilitate selective internalization of the drug into target cancer cells, enhance localized drug accumulation, reduce off-target effects (systemic toxicity), improve therapeutic efficacy, and overcome drug resistance [122]. In contrast, normal cell lines such as HSF typically exhibit only basal or negligible levels of the standard CD44 receptor isoforms [123], whereas the cancerous A-431 cell line is characterized by the robust overexpression of both standard and variant CD44 isoforms [124]. This large receptor density difference allows the HA-containing nanocomposite to be selectively targeted by malignant cells while limiting off-target effects on normal physiological tissues.

The results of this study are in strong alignment with other studies reporting that chitosan/HA nanocomposites can enhance targeted drug delivery in various tumor types, including oral squamous cell carcinoma [125], and skin cancer [126]. It is noteworthy that chitosan-based drug delivery systems can integrate both passive and active targeting strategies. Chitosan NPs can be first designed to accumulate in the tumor via the EPR phenomenon, enhance drug release through pH stimulus-responsiveness in the acidic tumor microenvironment, and then actively target specific cancer cells using surface-conjugated ligands. This multifunctional synergistic platform aims to optimize specific site drug localization and enhance internalization kinetics, hence potentiating the therapeutic index of AMG within the tumor microenvironment.

## Conclusion

Amygdalin (AMG) is a plant-derived cyanogenic glycoside with potent anticancer properties. However, its clinical application is usually limited due to poor specificity and systemic toxicity. In this study, a multifunctional quaternary nanocomposite (CS/rGO-ZnO/HA) was synthesized via ionic gelation and chemical cross-linking to encapsulate AMG. Drug-loaded nanocomposite exhibited an average diameter of 180–200 nm and a robust negative zeta potential of about − 36 mV, indicating high colloidal stability. In vitro release kinetics of AMG from the nanocomposite demonstrated a controlled, pH-dependent profile, with accelerated drug release under acidic biomimetic conditions. Moreover, in vitrocytotoxicity assay revealed that the AMG-loaded nanocomposite exerted enhanced cytotoxic effects against A-431 squamous cell carcinoma cells, while maintaining superior biocompatibility toward normal human skin fibroblast (HSF) cells. This improved selectivity, combined with synergistic anti-migratory activity and enhanced cellular uptake, suggests that this nanoplatform could serve as a promising candidate for head and neck cancer therapy. However, further mechanistic pathways need to be validated, including antioxidant potential, CD44 blocking, and enzymatic activation of AMG. Furtherin vivo investigations are in progress to validate the tumor-targeting efficiency and systemic safety profile of the developed nanocomposite prior to possible clinical translation. Moreover, further research is required to validate this nanocomposite as a suitable candidate for local transdermal or transmucosal microneedle-based delivery platforms.

## Supplementary Information

Supplementary Material 1

## Data Availability

The data utilized in the present study will be available upon request from the corresponding authors.

## Abbreviations

HNSCC: Head and Neck Squamous Cell Carcinoma
SCC: Squamous Cell Carcinoma
AMG: Amygdalin
DDSs: Drug Delivery Systems
CSNPS: Chitosan nanoparticles
rGO-ZnO: Reduced Graphene oxide doped with zinc oxide nanoparticles
HA: Hyaluronic Acid
NaOH: Sodium hydroxide
CS/rGO-ZnO/HA: Hyaluronic acid-coated chitosan Functionalized with rGO/ZnO NPs
CS/rGO-ZnO/HA/AMG: AMG -loaded nanocomposite
PBS: Phosphate-buffered saline
SEM: Scanning electron microscope
TEM: Transmission Electron Microscopy
PS: Particle size
DLS: Dynamic light scattering
ZP: Zeta potential
EE%: Entrapment efficiency percentage
PDI: Polydispersity Index
FTIR: Fourier transform infrared spectroscopy
XRD: X-ray powder diffraction
DSC: Differential Scanning Colorimetry
TGA: Thermogravimetric Analysis
MTT: 3-(4,5-dimethylthiazol-2-yl)-2,5-diphenyltetrazoliumbromide
DMSO: Dimethylsulfoxide
ROS: Reactive Oxygen Species
TME: Tumor Microenvironment
HMwt.: High molecular weight
LMwt.: Low molecular weight
ECM: Extracellular Matrix
EPR: Enhanced Permeability and Retention Effect
RES: Reticuloendothelial system
CSCs: Cancer Stem Cells
