# Supplementary material for: Targeted nanocomposite delivery system of amygdalin using chitosan/reduced graphene oxide-zinc oxide/hyaluronic acid for treatment of head and neck squamous cell carcinoma
Source: BMC Oral Health. 2026 Mar 25;26:627. doi: 10.1186/s12903-026-07943-1 (PMC13063543; doi:10.1186/s12903-026-07943-1)
Supplement: Supplementary file 1 — Supplementary Material 1 [file 12903_2026_7943_MOESM1_ESM.docx]

**Supplementary figures**

**Fig. S1.** DSC thermograms of blank (CS/rGo-ZnO/HA) nanocomposite, AMG-loaded (CS/rGo-ZnO/HA) nanocomposite, and individual components.


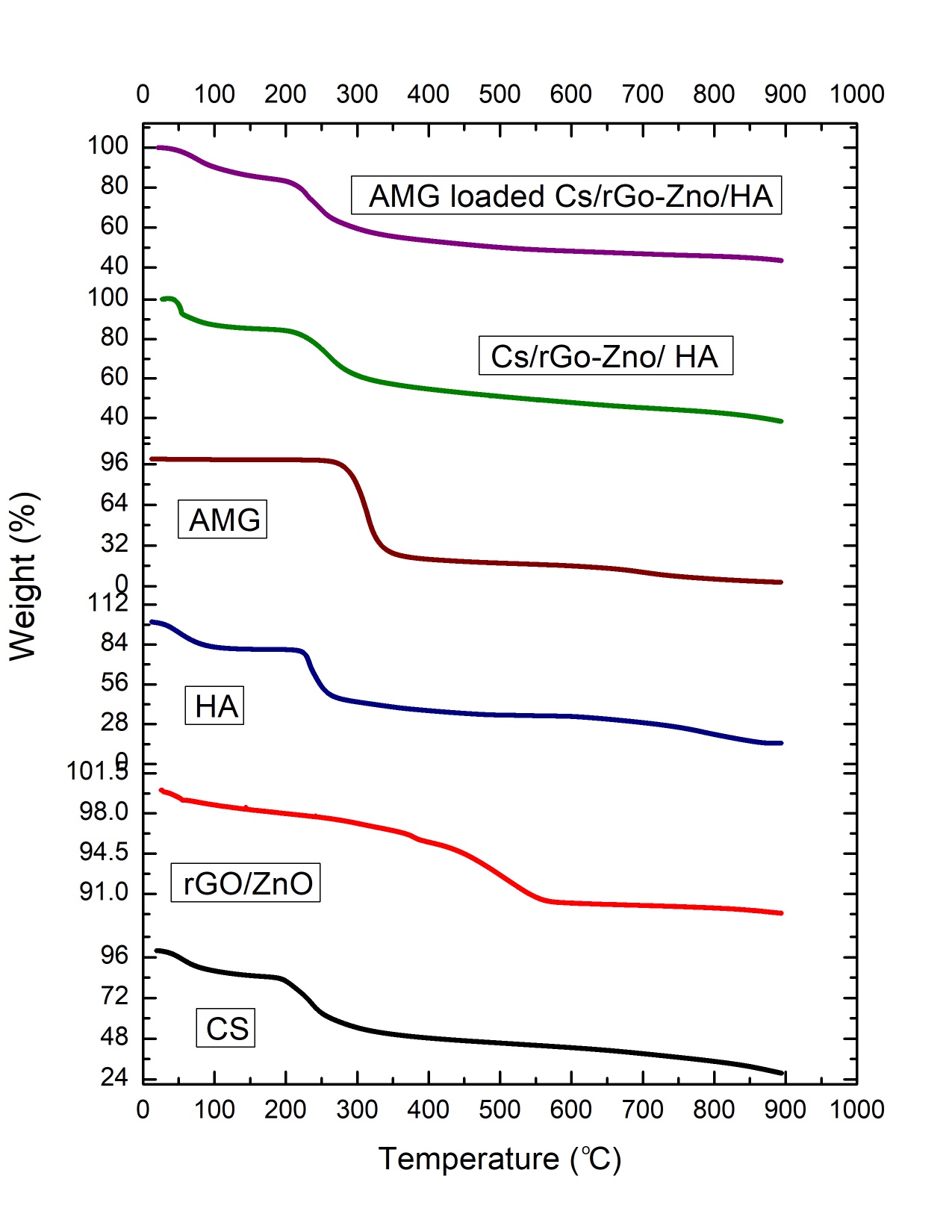


**Fig. S2.** TGA thermograms of blank (CS/rGo-ZnO/HA) nanocomposite, AMG-loaded (CS/rGo-ZnO/HA) nanocomposite, and individual components.
